# Feasibility and Effectiveness of the Exercise Program in Endometrial Cancer; Feasibility and Acceptability Survivorship Trial (EPEC-FAST)

**DOI:** 10.3390/cancers14225579

**Published:** 2022-11-14

**Authors:** Anke Smits, Khadra Galaal, Steve Winnan, Alberto Lopes, Ruud L. M. Bekkers

**Affiliations:** 1 Gynecological Oncology, Radboudumc, Geert Grooteplein Zuid 10, 6525 GA Nijmegen, The Netherlands; 2 Gynecological Oncology, Sultan Qaboos Comprehensive Cancer Care and Research Centre, Seeb 999046, Oman; 3 Royal Cornwall Hospital Trust, Penryn TR10 9FE, UK; 4 Gynecological Oncology, Royal Cornwall Hospital Trust, Truro TR1 3HD, UK; 5 Gynecological Oncology, Catharina Hospital Eindhoven, 5623 EJ Eindhoven, The Netherlands

**Keywords:** endometrial cancer, exercise, feasibility, quality of life, weight, fitness

## Abstract

**Simple Summary:**

Endometrial cancer has a relatively good prognosis, resulting in a growing number of long-term survivors. Quality of life is an important outcome for cancer survivors. However, many survivors report impaired quality of life due to obesity and a sedentary lifestyle. In this trial, we assessed an individualized exercise program for endometrial cancer patients aimed to improve quality of life and other health outcomes including weight and physical fitness. We showed that our individualized one-to-one exercise intervention in endometrial cancer patients is feasible in terms of patient recruitment, execution and safety. The program resulted in significantly improved quality of life, weight and physical fitness of patients. Future studies need to further assess these effects on quality of life and other health outcomes including anthropometrics and survival.

**Abstract:**

To evaluate the feasibility of an individualized exercise program in the standard care for endometrial cancer patients aimed to improve quality of life and other health outcomes. This was a single-arm prospective intervention trial to assess the feasibility of an individualized exercise intervention in endometrial cancer patients after treatment. The exercise intervention consisted of weekly individualized training sessions, for 10 weeks, at a local gym facility. The program started six weeks post-operatively. Primary outcomes were feasibility aspects including number of eligible patients, recruitment and adherence rates. Secondary outcomes included quality of life outcomes and anthropometric measures. A total of 54 women were eligible for participation, of which 22 (41%) consented to the study. Overall attendance was 86%, and there were no adverse events. There was a significant improvement in quality of life outcomes, including role (*p* = 0.02), emotional (*p* = 0.02) and cognitive functioning (*p* = 0.04). In addition, there was a significant improvement in visceral fat percentage (*p* = 0.039) and physical fitness (six-minute walk test *p <* 0.001). The maximum weight loss achieved was 6.0 kg after 3 months and 8.4 kg after 6 months. An individualized one-to-one exercise intervention in endometrial cancer patients is feasible in terms of recruitment, adherence and safety.

## 1. Introduction

Endometrial cancer affects more than 9000 women annually in the United Kingdom [1]. The relatively good prognosis has resulted in an increasing group of long-term survivors, with over 70,000 survivors in the United Kingdom alone [1]. Consequently, survivorship care including quality of life outcomes have become an integral part of cancer care.

The majority of endometrial cancer patients are overweight or obese and do not meet current recommendations for physical activity [2,3]. It has been well established that increased body mass index (BMI) and impaired physical activity levels are associated with impaired quality of life, surpassing the negative effects of endometrial cancer diagnosis and treatment alone [2,3,4,5,6]. In addition, obese endometrial cancer patients are at risk of numerous obesity-related comorbidities, with cardiovascular disease being the leading cause of death in their survivorship era [7,8,9,10].

Exercise and weight reduction have been identified as a means to improve the quality of life and health-related outcomes of cancer patients. However, addressing this in the endometrial cancer population has proven to be a significant challenge, specifically for older survivors (aged >65 years) [11,12]. Most studies have focused on demonstrating feasibility of home-based or unsupervised interventions but have so far failed to demonstrate the necessary improvements in weight, physical activity and quality of life [13,14,15,16]. The effect of supervised exercise interventions for women have not been extensively assessed, despite these patients expressing a specific interest in one-to-one exercise sessions within 6 months after treatment [17,18].

We therefore undertook an evaluation for the feasibility of introducing an individualized and supervised exercise program at a local gym facility into the treatment phase of care for endometrial cancer patients aimed to improve the post-treatment quality of life and other health outcomes.

## 2. Methods

This was a single-arm prospective intervention trial to assess the feasibility of an individualized exercise intervention for endometrial cancer patients within the current clinical care pathway. The protocol of the study has previously been described, and has been approved by the Exeter National Research Ethics Service (NRES) Committee [7]. The study took place at the Royal Cornwall Hospital Trust (RCHT). The RCHT is the main hospital of Cornwall, a rural county in the south west of England with a dispersed population of 568,210 in the census of 2022 [19]. In addition, Cornwall’s population is getting older as average life expectancy continues to rise. Trial Registration Number: NCT02367950.

### 2.1. Study Population and Recruitment

Eligible participants were women with a diagnosis of primary endometrial cancer undergoing treatment with curative intent. We excluded women who (1) were aged <18 years, (2) had a concurrent cancer diagnosis, (3) had an Eastern Cooperative Oncology Group (ECOG) performance score of >2, or (4) were unable to give informed consent. As this was a feasibility study, no calculation for sample size was performed.

Women attending the gynecological outpatient clinic were evaluated for eligibility. Potentially eligible women were identified prior to surgery and were given an information leaflet of the study. Eligible women were recruited post-treatment after receiving final histological diagnosis, further treatment and follow-up plan. The intervention was started after their six-week follow-up appointment after surgery or after completing adjuvant therapy. The recruitment period was from January 2015 until November 2015.

### 2.2. Intervention

The exercise intervention consisted of 60 min individualized (one-to-one) training sessions with a personal trainer, once a week, for 10 consecutive weeks. The program started after the patients’ six weeks post-operative check-up and took place at a local gym facility. The program was tailored to the individual patient through a health assessment which took into consideration their current health status, physical activity level, comorbidities and medical history. Each session consisted of a 10 min warm-up, a 40 min workout of a combination of aerobic exercise (cardiovascular), pillar strength exercise (including hip and core stability) and resistance training, and 10 min cool down, and was instructed by the personal trainer. The content and duration of the intervention was based on national and cancer-specific recommendations of the American College of Sports Medicine, evidence from the literature, and feedback from relevant patient groups [20,21,22]. A detailed description of the theory-based intervention has been described previously, and a layout of the sessions is detailed in Table 1 [7]. The exercise phase was performed at a level of 40–60% maximum heart rate, measured with a Polar Heart Rate monitor using the Karvonen method for calculating the target heart rate interval. Resistance training was performed at an intensity of 40–60% with one repetition maximum (1 RM). Participants also received general physical activity recommendations for moderate intensity exercise for 150 min per week as part of standard practice [21,23].

### 2.3. Outcomes

The primary outcomes of the study were feasibility aspects including number of eligible patients, recruitment rates and willingness of clinicians to recruit. Adherence rates to the exercise program in terms of attendance was also included as primary outcome and were collected from registration forms used during the exercise program. Further outcomes included barriers to participation and adverse events related to the program.

Secondary outcomes comprised quality of life outcomes which were evaluated through the international European Organization for Research and Treatment of Cancer Quality of Life Questionnaire-Core 30 (EORTC) QLQ-C30 questionnaire at baseline, three and six months after the intervention. The QLQ-C30 is a cancer-specific questionnaire covering global quality of life, several other areas including physical, role, emotional, cognitive and social functioning [24].

Other secondary outcomes were anthropometric measures which were collected at baseline and after completion of the program (three months and six months). These measures included weight, BMI, body fat percentage, lean muscle tissue and resting metabolism, measured with a body composition monitor and the six-minute walk test. The six-minute walk test was performed on a treadmill, using an adaptation of the American Thoracic guidelines [25].

### 2.4. Statistical Analysis

Statistical analyses were performed using SPSS statistical software, Version 24.0 [26]. Continuous outcomes were presented as means and standard deviations, and categorical data were presented as frequencies and proportions. Outcomes of the EORTC QLQ-C30 were linearly transformed to 0–100 scores. Data were compared using the paired sample *t*-test and the repeated measures ANOVA for continuous data. Study results have been reported in adherence to the CONSORT 2010 guidelines [27].

## 3. Results

### 3.1. Recruitment

Sixty women were diagnosed with endometrial cancer between January 2015 until November 2015, of which 54 were eligible and approached for participation. A total of 22 women agreed to participate in the study, resulting in a recruitment rate of 40.7%.

Four women initially recruited, withdrew from the study before the first session because of travel issues, unwillingness to complete questionnaires or already being adequately active. Reasons for declining participation in the study were documented in most women approached (87.5%). The most frequently stated reasons were travel and transportation problems (62.5%), not wanting to complete questionnaires (9.4%), being elderly (9.4%) or already having a good physical activity level (3.1%).

The gynecological oncology team at the RCHT was supportive of the trial and did not raise any concerns about the study prior to commencement nor during the study period.

### 3.2. Patient Demographics and Clinical Characteristics

The eventual study population comprised 18 women (33.3% of all women approached). Baseline characteristics of the study population are shown in Table 2. Median age was 62 years, with the oldest woman participating aged 82 years old. Five women (27.8%) in the study population were 70 years or older and 12 (66.7%) were obese with a BMI of ≥30 kg/m^2^. Overall, 17 women (94%) were Caucasian, 15 women had an ECOG performance status of 0, and 16 women had one or more comorbidities. Most prevalent comorbidities were hypertension (N = 7), diabetes mellitus (N = 4), asthma (N = 3) and osteoarthritis (N = 2). All women were diagnosed with endometrioid adenocarcinoma stage 1, with grade 1 (72.2%) or grade 2 (27.8%) histology. A total hysterectomy (abdominal approach or laparoscopic approach) with bilateral salpingo-oophorectomy was performed, except for one woman who requested one ovary to be left in situ. Two women received adjuvant radiotherapy treatment, for which the exercise program was delayed until completion of adjuvant treatment.

### 3.3. Adherence

During the study, one woman dropped out after two sessions, because of scheduling and travel issues. Overall attendance was 86%. The average number of sessions completed by the remaining 17 women was nine, with eleven women completing all 10 sessions. Reasons for non-attendance were travel and transportation problems, family issues or problems with scheduling. Difficulties in delivering the interventions were mainly scheduling issues as we chose not to assign a specific day for the program but rather scheduled this according to the participants’ availability. However, no session was missed because of scheduling problems.

One woman developed a back problem at work and was unable to continue beyond four sessions and another participant discontinued after seven sessions because of an arm infection with consequent lymphoedema having had an axillary lymph node resection for breast cancer four years previously. This was not a consequence or due to an injury of the exercise program. All women completed their follow-up assessments as these coincided with their clinical appointments. No adverse events of the program were reported by patients or personal trainer.

### 3.4. Quality of Life

Mean outcomes of the quality of life domains of the whole population are illustrated in Table 3. Overall, there was an improvement in reported outcomes over time, with the greatest increase seen in the first post-intervention assessment. Role, emotional and cognitive functioning showed a significant difference over time (*p* = 0.02, *p* = 0.02, *p* = 0.04). Two women did not complete the six-month follow-up questionnaires and were excluded from the analysis. No adverse events of the program were reported by patients or personal trainer.

### 3.5. Anthropometric Measures

Outcomes in terms of anthropometric measures are illustrated in Table 4. Women participating had an initial mean weight of 86.0 kg. There was a slight decrease in both weight and BMI immediately after the intervention which persisted three months after the intervention. The maximum reduction in weight achieved was 6.0 kg after 3 months (post-intervention) and 8.4 kg after 6 months. There was a decrease in average visceral fat percentage of 0.3% (*p* = 0.039). In addition, there was a significant increase in the distance walked during the six-minute walk test (*p* < 0.001).

## 4. Discussion

Approximately two million people in England are currently living after a diagnosis of cancer. This number is expected to increase by 2% annually, and the total number of cancer survivors is projected to rise to over 3 million by 2030 [28]. It is essential to identify key interventions that could impact health related outcomes and quality of life of cancer survivors, as they are struggling to maintain or improve their physical activity levels [29]. Physical activity programs and healthy weight management have been proposed as key interventions [30]. This is one of the first studies to demonstrate the feasibility of a supervised individualized exercise program at a local gym facility in the treatment phase of care of endometrial cancer patients, aimed to improve quality of life and anthropometric measures.

All participants were recruited on-site resulting in a recruitment rate of 40.7%. This is high compared to other endometrial cancer lifestyle intervention studies which have rates varying from 18.9% to 29.9% [31,32,33,34,35]. We attribute this to the on-site face-to-face recruitment, rather than mail or telephone invitations, which have been shown to be less effective [35]. We recruited patients within the treatment phase of care, as women prefer to start within 6 months of completion of treatment and are at a ‘teachable moment’, willing to modify their lifestyle behavior to achieve improved health [36,37].

The 86% adherence of our study is among the highest reported for a lifestyle intervention for endometrial cancer patients. Our high attendance rates may be explained by our individualized one-to-one sessions and the accessibility of a local gym, as they are known factors to improve exercise engagement and overcome barriers [17,38]. Studies by Rossi et al. and Koutoukidis et al. evaluating face to face interventions delivered through group sessions have reported rates of 60 to 77% [31,35]. Other home-based interventions which evaluated telephone counselling or mobile applications also reported lower adherence rates [13,15,32,34]. However, the large variation in recruitment strategies, eligibility criteria, program components and duration preclude direct comparisons.

Our study showed a significant increase in several aspects of quality of life after completing the exercise intervention, including emotional, cognitive and role functioning. Despite being preliminary results, other studies have not reported these improvements [13,16,39,40]. Interestingly, physical functioning did not significantly increase after the intervention, while other lifestyle intervention studies have reported significant improvements [33,34]. In addition, global quality of life did not show a significant improvement after completion of the exercise program, mirroring the findings of a systematic review on lifestyle interventions in endometrial cancer we previously published [13]. However, following the single-arm design and the study size, large controlled studies are needed to further assess these preliminary findings against the natural progression of quality of life post-treatment (control group), the long-term effects of the intervention on quality of life. In addition, we recommend more extensive assessment of quality of life domains through different questionnaires in future trials.

The current study showed a significant improvement of physical fitness over time, and a possible reduction in visceral fat. However, as our study is only a feasibility study with a relatively small sample size and no control group, no conclusions can be drawn as to the (long-term) effectiveness and sustainability of the trial, and this should be evaluated by future trials. However, previous studies support that lifestyle interventions result in significant weight loss and improved physical activity levels among endometrial cancer patients [13,35,41]. Interestingly, studies by Zamorano et al. and Rahimy et al., assessing a text-message or telephone-based programs, showed no significant weight loss or sustained physical activity improvements among endometrial cancer survivors [39,42]. In addition, cost-effectiveness is also an important factor which must be considered. Alternatives such as low-cost online interventions have the potential to have a broad reach but may not directly translate to improved effectiveness and sustainability and therefore need to be an important focus of future research [39,42].

This study has demonstrated the feasibility of an exercise program that can be adopted easily within existing clinical care pathway for endometrial cancer patients. This allows for on-site recruitment and decreases the burden for patients as they do not need to undertake additional clinical visits. We believe the timing of our invention is also an important strength and paramount to its success as it specifically adheres to the preferences of our patients. Importantly, we included a significant amount of older cancer patients, as they are usually underrepresented in exercise-based trials [43]. Another strength of the intervention is the one-to-one individualized sessions, in which our population has expressed a specific interest, with individual guidance and attainable goals being known to further enhance adherence to an intervention [17,18,22].

Unfortunately, the generalizability of the outcomes is limited by the sample size and the setting. The trial was held in a rural area with a dispersed population. This caused travelling to be an issue and therefore a major barrier to participation, which has also been demonstrated by other studies [31,44]. Recruitment would probably have been significantly higher if there had been more intervention sites, as 37% of women declined because of the distance involved. Future recruitment may be aided by travel reimbursements and decreasing the travel distance. Other reported barriers included the use of questionnaires in the outcomes and women perceiving themselves too old for exercise. Women with a variety of ages were recruited, and therefore, further reduction in this barrier is anticipated through appropriate patient education. Individual and disease-specific barriers such as inconvenience, tiredness, and feeling unwell have also been described, but were not commonly reported in our study [17,31]. In addition, we have deemed the program safe, with no adverse events due to the program being reported by the exercise trainer or participants.

Another important limitation of the study was that the effect of the intervention on overall physical activity behavior was not assessed. In addition, we did not assess pre-diagnosis exercise behavior and recreational physical activity during and after the program. These are known factors that may influence outcomes of the intervention [5,17,45]. Friedrich et al. found that postdiagnosis recreational physical activity was strongly associated with improved disease-free and overall survival in endometrial cancer patients, which emphasizes the importance of a postdiagnosis exercise program [46]. We believe should be assessed and included as an outcome in a definitive trial, through self-reported or objective measures such as accelerometers. In addition, we recommend assessing anthropometric outcomes beyond the six-month follow-up, to evaluate the sustainability of changes in outcomes.

## 5. Conclusions

With this study, we have demonstrated the feasibility of an individualized one-to-one exercise intervention in endometrial cancer patients in terms of recruitment, adherence and safety. Our program fits well into the current care pathway and adheres to patient preferences. Travel and transportation were the main barriers to participation and adherence, and further attempts to reduce this barrier should be made. The results of the feasibility trial will form the basis for a randomized controlled trial.

## Figures and Tables

**Table 1:** The EPEC-FAST Exercise Program.

Exercise Phase *	Details	
Warm-Up		
10 min	Low-intensity warm-up using an exercise bike or a treadmill	
Exercise phase		
40 min	Aerobic exercise (20 min) Walking on a treadmill or cycling on an exercise bike. The exercise phase will be performed at a level of 40–60% of maximum heart rate.	
	Pillar strength training (10 min) Consists of 4 exercises to improve stability and strength of the hip, and 3 exercises to improve core stability and strength. Patients are recommended to perform 8 repetitions of each of the hip stability movements per leg, and a set of 10–15 repetitions of each core muscle exercise. A stability ball may be used to facilitate some of the exercises	
	Hip movements: - Hip flexion - Hip extension - Hip extension - Hip adduction - Hip abduction	Core movements: - Crunch - Back - Opposite arm/leg raise
	Resistance training (10 min) Consists of 1 set of 8 to 12 repetitions of 8 exercises that include all the major muscle groups. After initial phase of repetitions, this can be increased up to 20–25 repetitions (40–60% of 1 RM) during 1 session. A dumb-bell, stability ball or bench may be used to facilitate the exercises. Exercises: - Basic squat - Lateral raise - Dumb-bell deadlift - Shoulder press - Hamstring curl - Dumb-bell biceps curl - Overhead triceps extension - Calf raise	
Cool down		
10 min	Set of 6 stretching and flexibility exercises. Four repetitions of each of the following muscle groups will be performed for 10–30 s. - Lower back - Tensor fasciae latae - Hip flexor - Quadriceps - Hamstring - Calf	

* The content of the program will subject to individual variability and will be adjusted to the individual patient.

**Table 2:** Characteristics of the study population.

Demographic Characteristics	Number (%)
Age (median, range)	62 (45–82)
BMI	
18.5–24.9 kg/m^2^	1 (5.6%)
25–29.9 kg/m^2^	5 (27.8%)
30–39.9 kg/m^2^	9 (50.0%)
≥40 kg/m^2^	3 (16.7%)
Ethnicity	
White	17 (94.4%)
Other	1 (5.6%)
ECOG performance status	
0	15 (83.3%)
1	3 (16.7%)
Comorbidities	
None	2 (11.1%)
One	6 (33.3%)
Two or more	10 (55.6%)
Smoking	
Yes	0 (0%)
No	18 (100%)
Stage	
IA	14 (77.8%)
IB	4 (22.2%)
Grade	
1	13 (72.2%)
2	5 (27.8%)
3	0 (0%)
Operation	
TLH + BSO	15 (83.3%)
TAH + BSO	2 (11.1%)
Other	1 (5.6%)

BSO: bilateral salpingo-oophorectomy; BMI: body mass index; ECOG: Eastern Cooperative Oncology Group; TAH: total abdominal hysterectomy; TLH: total laparoscopic hysterectomy.

**Table 3:** Quality of life outcomes over time.

	Baseline	Post-Intervention	6 Months	Analysis
	N = 18	N = 18	N = 16	
	Mean (Standard Deviation)			*p*-Value
EORTC QLQ-30				
Global quality of life	73.0 (18.3)	82.9 (19.5)	82.8 (15.3)	0.12
Physical functioning	86.7 (12.8)	92.6 (11.2)	92.5 (10.6)	0.06
Role functioning	76.9 (25.0)	97.2 (6.4)	90.6 (18.2)	0.02 *
Emotional functioning	80.9 (19.5)	92.1 (11.9)	86.5 (18.5)	0.02 *
Cognitive functioning	84.3 (19.1)	90.7 (11.7)	91.7 (13.6)	0.04 *
Social functioning	86.3 (23.0)	97.2 (6.4)	95.8 (9.6)	0.1

*: *p <* 0.05.

**Table 4:** Anthropometric outcomes over time.

	Baseline	Post-Intervention	Six Months	Analysis
	N = 18	N = 18	N = 18	
	Mean (Standard Deviation)			*p*-Value
Weight	86.0 (16.7)	85.3 (16.8)	85.0 (17.1)	0.212
BMI	33.0 (6.5)	32.7 (6.4)	32.6 (6.5)	0.194
Body fat percentage	44.6 (6.9)	42.4 (7.4)		0.08
Visceral fat percentage	11.6 (3.1)	11.3 (3.1)		0.039 *
Six-minute walk test (km)	0.383 (0.125)	0.535 (0.101)		<0.001 *

*: *p <* 0.05.

## Data Availability

Not applicable.
